# Supplementary material for: Multisite Injections of Canine Glial-Restricted Progenitors Promote Brain Myelination and Extend the Survival of Dysmyelinated Mice
Source: Int J Mol Sci. 2024 Oct 1;25(19):10580. doi: 10.3390/ijms251910580 (PMC11477205; doi:10.3390/ijms251910580)
Supplement: Supplementary file 1 [file ijms-25-10580-s001.zip › ijms-3128281-supplementary.pdf]

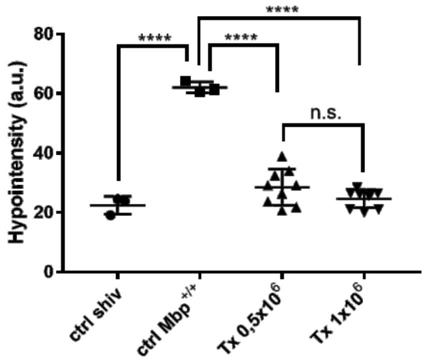

Supplementary figure 1. Hypomyelination level in the corpus callosum of control shiverer mice (n = 4), control Mbp$^{+/+}$ mice (n = 3), and transplanted shiverer mice (tx 0,5x10^{6} n=10, tx 1x10^{6} n=10). No statistically significant differences were observed between transplanted groups. Abbreviations: a.u., arbitrary units; Mbp, myelin basic protein, n.s. – not significant.

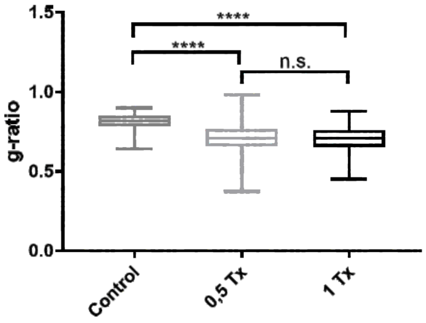

Supplementary figure 2. Analysis of myelin thickness on transmission electron microscopy images presented as a g-ratio. The myelin surrounding the axons of transplanted animals n=8 (tx 0,5x10^{6} n=5, tx 1x10^{6} n=3) is significantly thicker than myelin in non-transplanted animals (n=4) as compared by g-ratio analysis. ****p<0,0001, n.s. – not significant.

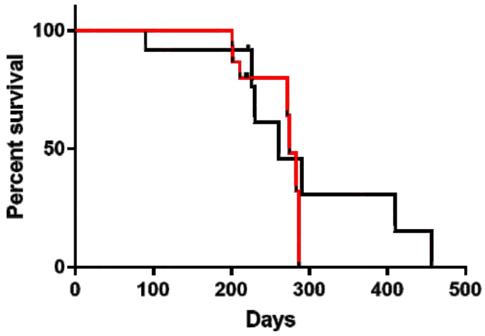

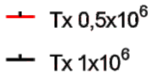

Supplementary figure 3. Mice lifespan analysis is depicted as a Kaplan-Meier curve. A survival curve was created for n = 15 of Tx 0.5x10^{6} and n = 12 Tx 1x10^{6} transplanted animals. Survival curve analysis reflects a lack of difference in survival of mice transplanted with different amounts of cells. Log rank Matel Cox test p-value – ns. , Gehan-Breslow-Wilcoxon test p-value – ns. The median survival of the Tx 0.5x10^{6} group was 274 days whereas in the case of the Tx 1x10^{6} – 261 days.

EXP1		
cGRPs Ki67(g)+D_20x_a		
DAPI+	Ki67+	
25	5	20%
cGRPs Ki67(g)+D_20x_b		
DAPI+	Ki67+	
30	5	17%
cGRPs Ki67(g)+D_20x_c		
DAPI+	Ki67+	
15	3	20%
EXP2		
cGRPs Ki67(g)+D_20x_d		
DAPI+	Ki67+	
43	5	12%
cGRPs Ki67(g)+D_20x_e		
DAPI+	Ki67+	
41	7	17%
cGRPs Ki67(g)+D_20x_f		
DAPI+	Ki67+	
31	5	16%
EXP3		
cGRPs Ki67(g)+D_20x_g		
DAPI+	Ki67+	
29	4	14%
cGRPs Ki67(g)+D_20x_h		
DAPI+	Ki67+	
39	7	18%
cGRPs Ki67(g)+D_20x_i		
DAPI+	Ki67+	
28	6	21%
	avg	17%

Supplementary information: The number of proliferative cells (Ki67+) analyzed by immunocytochemical staining. Cells were photographed under 10× lens, and counted manually in a blind fashion. Three regions of interest (ROIs) were selected from two separate culture plate wells. This process was repeated across three independent biological replicates.
